# Nasopharyngeal bacterial load as a marker for rapid and easy diagnosis of invasive pneumococcal disease in children from Mozambique

**DOI:** 10.1371/journal.pone.0184762

**Published:** 2017-09-14

**Authors:** Pedro Brotons, Quique Bassat, Miguel Lanaspa, Desiree Henares, Amaresh Perez-Arguello, Lola Madrid, Reyes Balcells, Sozinho Acacio, Maria Andres-Franch, Maria Angeles Marcos, Ana Valero-Rello, Carmen Muñoz-Almagro

**Affiliations:** 1 Molecular Microbiology Department, Institut de Recerca Sant Joan de Déu, University Hospital Sant Joan de Déu, Barcelona, Spain; 2 CIBER de Epidemiología y Salud Pública CIBERESP, Instituto de Salud Carlos III, Madrid, Spain; 3 ISGlobal, Barcelona Ctr. Int. Health Res. (CRESIB), Hospital Clínic - Universitat de Barcelona, Barcelona, Spain; 4 Centro de Investigação em Saúde de Manhiça (CISM), Maputo, Mozambique; 5 ICREA, Pg. Lluís Companys 23, Barcelona, Spain; 6 Pediatric Infectious Diseases Unit, Pediatrics Department, Hospital Sant Joan de Deu (University of Barcelona), Barcelona, Spain; 7 Universidad Europea de Madrid, Madrid, Spain; 8 School of Medicine, Universitat Internacional de Catalunya, Barcelona, Spain; Universidade de Lisboa Faculdade de Medicina, PORTUGAL

## Abstract

**Background:**

Current diagnostic methods for detection of *Streptococcus pneumoniae* in children with suspected invasive pneumococcal disease have limitations of accuracy, timeliness, and patient convenience. This study aimed to determine the performance of pneumococcal load quantified with a real-time polymerase-chain reaction in nasopharyngeal samples to diagnose invasive pneumococcal disease in children.

**Methods:**

Matched case-control study of patients <5 years of age with invasive pneumococcal disease admitted to the Manhiça District Hospital (Mozambique) and asymptomatic controls recruited in different periods between 2006 and 2014. Cases were confirmed by a positive bacterial culture for *S*. *pneumoniae* in blood or cerebrospinal fluid. Nasopharyngeal aspirates were collected from cases and controls and pneumococcal density was quantified by *lytA* real-time polymerase-chain reaction.

**Results:**

Thirty cases (median age 12.8 months) and sixty controls (median age 11.7 months) were enrolled and 70% of them were male. Nasopharyngeal pneumococcal carriage was high in both groups: 28/30 (93.3%) for cases *vs*. 53/60 (88.3%) for controls (*p* = 0.71). Mean nasopharyngeal pneumococcal load was identified as a marker for invasive pneumococcal disease (7.0 log_10_ copies/mL in cases *vs*. 5.8 log_10_ copies/mL in controls, *p*<0.001) and showed good discriminatory power (AUC-ROC: 82.1%, 95% CI 72.5%-91.8%). A colonization density of 6.5 log_10_ copies/mL was determined as the optimal cut-off value to distinguish cases from controls (sensitivity 75.0%, specificity 73.6%).

**Conclusion:**

Use of non-invasive nasopharyngeal aspirates coupled with rapid and accurate quantification of pneumococcal load by real-time polymerase chain reaction has the potential to become a useful surrogate marker for early diagnosis of invasive pneumococcal disease in children.

## Introduction

The gram-positive bacterium *Streptococcus pneumoniae* (*S*. *pneumoniae*), a frequent colonizer of the child’s nasopharynx, remains one of the major killers of children globally despite extensive pneumococcal vaccination programs [1–3]. Occasionally, colonizing pneumococci spread from their nasopharyngeal (NP) niche to normally sterile body sites and cause potentially life-threatening invasive pneumococcal disease (IPD) [4]. The complex mechanisms underlying the transition from NP colonization to IPD are far from being fully understood, although a number of environmental, host, and pathogen-related risk factors have been identified [5–8].

Diagnosis of IPD remains challenging in children, as it requires collection of invasive samples and the use of sensitive methods to detect *S*. *pneumoniae* in small sample volumes [9]. Culture-based methods on sterile fluids are insensitive and time-consuming while rapid, simple, non-invasive pneumococcal antigen detection tests on urine have become the mainstay for the screening of pneumococcal infections in adults but show poor specificity in children [10]. Relatively quick real-time polymerase-chain reaction (PCR) assays such as those targeting the *lytA* gene accurately detect *S*. *pneumoniae* in small volumes of both invasive and non-invasive pediatric samples [11], but are not easily accessible and feasible. In addition, since IPD and many other common pediatric conditions share overlapping clinical presentation [12], empirical diagnosis without microbiological confirmation can result in antibiotic misuse and increased antimicrobial resistance in the community [13].

NP pneumococcal load, measured by real-time PCR or culture in non-invasive samples collected from the upper respiratory tract, has been proposed as a candidate surrogate marker to discriminate IPD from asymptomatic carriage, albeit with conflicting evidence [14–20]. In this study, we aimed to determine whether pneumococcal load quantified with a *lytA* real-time PCR in NP aspirates taken from a rural Mozambican pediatric population could be a marker of IPD, while also taking into account other diverse pathogen- and host-related potential risk factors.

## Methods

### Study design, site and population

This case-control study was conducted at the Manhiça District, Southern Mozambique, between 2006 and 2014. The *Centro de Investigação em Saúde de Manhiça* (CISM) has been running a Demographic Surveillance System (DSS) in the area since 1996 and a morbidity surveillance system at Manhiça District Hospital (MDH), covering an area at the time of the study of around 500 km^2^, with ~92,000 people under permanent surveillance, 19% of which less than 5 years of age [21]. The rural area of Manhiça is co-endemic for malaria, and severely affected by the Human Immunodeficiency Virus (HIV) pandemic [22]. The ten-valent pneumococcal conjugate vaccine was introduced in Mozambique in March 2013.

### Selection of cases and controls. Data collection

Cases of IPD were prospectively selected among inpatients <5 years of age recruited as part of invasive bacterial disease and pneumonia clinical studies at MDH [23,24] during the period September 2006-May 2014, under a signed informed consent of their mothers or legal guardians. Before the initiation of treatment, a NP aspirate was collected for both cases and controls to determine pneumococcal load and respiratory viral infection. Venous blood was collected for blood culture, full blood cell count, HIV and malaria testing.

Two asymptomatic controls for IPD of the same age (+/-3 months) and sex were randomly selected in the community for each case from the DSS databases, and recruited during the period September-November 2012, which span over both the hot rainy season and the dry cooler season. A study clinician confirmed they were healthy prior to recruitment.

Variables recorded in cases and controls included patient age, sex, height, weight, weight-for-age z (WAZ) score, nutritional status, recruitment season, co-infection with HIV or malaria, NP carriage (*lytA* positivity), NP pneumococcal load among *lytA* positive samples, pneumococcal serotype identification, serotype invasiveness potential, presence of other co-colonizer pneumococci and respiratory viruses in the nasopharynx, and blood marker measurements upon recruitment to the study. Children having received antibiotics in the previous month were excluded from the study.

### Definitions

IPD was defined as isolation of *S*. *pneumoniae* in blood or cerebrospinal fluid by culture. Nutritional status was established according to weight-for-age z-scores [25] and children with a weight-for-age z-score with a dispersion < -1SD were considered to be undernourished. The hot rainy season was defined as November to April and the dry cooler season as May to October. Serogroups 1, 5 and serotype 7F/A were considered to have high invasive disease potential as previously described [26].

### Nasopharyngeal sample collection

A NP aspirate method was selected as the best procedure for reducing discomfort of infants and young children during sampling. NP specimens were collected using NPAK^®^ kits (M-Pro, Michigan, US). They were processed into aliquots (obtained by injecting a small 2–3 mL amount of physiological saline, and then aspirating) and immediately frozen at -80°C. Frozen samples were shipped to Hospital Sant Joan de Déu and Hospital Clinic, both in Barcelona (Spain), where pneumococcal and respiratory virus studies were conducted, respectively.

### Laboratory methods

#### Detection and quantification of *S*. *pneumoniae*

Samples and standard reference control strains were extracted and concentrated by NucliSENS^®^ EasyMag^®^ (bioMérieux, Marcy l'Etoile, France) from an initial sample volume of 400 μL to an elution volume of 110 μL. Five micro liters of the DNA extract were added to the PCR reaction mix. A duplex real-time PCR targeting the *lytA* gene of *S*. *pneumoniae* and the internal control targeting RNaseP of human cells was performed. Sequence of primers and probes recommended by CDC for both the pathogen and the internal control were used (http://www.cdc.gov/meningitis/lab-manual/chpt10-pcr.html). DNA was amplified with the Applied Biosystems 7500 real-time PCR System (Applied Biosystems, CA, US).

#### Determination of PCR efficiency and the calibration curve for DNA quantification

A calibration curve correlating DNA pneumococcal load with cycle threshold value was performed by using reference strain *S*. *pneumoniae* R6. Given that genome-copy number is less variable than colony forming unit (CFU) for establishing standards [27], loads were quantified in genome-copy number which is about 2-logs higher than CFU. The calibration curve was generated by extracting the genomic DNA from an original suspension of *S*. *pneumoniae* R6 strain (OD_595_ = 0.5) and performing 10-fold serial dilutions that ranged from 10^8^ to 10^2^ copies per mL (cp/mL).

#### Pneumococcal serotyping

Capsular typing of pneumococci was directly performed in *lytA* positive samples without culture by multiplex PCR combined with automated fluorescence capillary electrophoresis according to a previous method published by our group [28]. Other non-detectable serotypes by this technique were classified as indistinguishable serotypes and were considered non-invasive for studying invasiveness potential. Patients with indistinguishable serotypes were excluded from the analysis of pneumococcal co-colonization as it was not possible to assess the number of non-identified colonizing serotypes in those subjects

#### Detection of respiratory viruses

Detection of influenza A, B, C, respiratory syncytial virus, adenovirus, coronavirus, enterovirus, human parainfluenza viruses 1, 2, 3, 4, human rhinovirus and human metapneumovirus was performed according to previously published studies [29,30].

#### Determination of HIV and malaria status

HIV-1 serodiagnosis was performed using a sequential testing algorithm with two rapid HIV-1 antibody tests (Determine^®^ and Unigold^®^). HIV-infection was confirmed when necessary by an HIV-1 DNA Amplicor test (version 1.5, Roche Molecular Systems, Inc., Branchburg, NJ, US) while thick and thin blood films were processed and examined according to standard methods for malaria diagnosis [31].

### Statistical analysis

Dichotomous variables were examined with the Chi-square test or the Fisher’s exact test. Continuous variables were described as mean (standard deviation, SD) or median values (interquartile range, IQR) and were compared using the t test for normal distributions or the Mann Whitney test for skewed data. The variable of NP pneumococcal load (copies/mL) was log transformed before inclusion in the analysis to assume a normal distribution. A preliminary comparison of cases and controls was conducted to ensure that a number of factors of interest were not differently distributed across groups. Logistic regression univariate and multivariate analyses were performed to identify risk factors for IPD. Variables that were found to be significantly associated with IPD in the univariate analysis were entered into multivariate models. The area under the receiver operating characteristic curve (AUC-ROC) was the parameter used to select the most accurate predictive model and determine the optimal cut-off value of NP pneumococcal load that maximizes sensitivity and specificity. Statistical significance was set at a p-value of <0.05 and confidence intervals (CI) at 95% level. All analyses were performed using Stata v.13 software (Stata Corp., College Station, TX, US).

### Ethical approval

The different surveillance and biomarker studies from which cases and healthy controls were drawn were approved in Mozambique by the Mozambican National Bioethics committee (Refs: 98/CNBS/06; 262/CNBS/10 and 228/CNBS/12) and in Spain by the Ethics Committee of the Hospital Clinic in Barcelona.

## Results

### Demographic characteristics of cases and controls

A total of 30 IPD patients and 60 healthy controls were included in the study. Males predominated in both groups (70%). The difference in median age between cases (12.8 months) and controls (11.7 months) was minor (*p* = 0.37). Twenty-five cases (83.3%) and all controls were recruited before the introduction of the pneumococcal conjugate vaccine in the country. Recruitment was not influenced by seasonality: 16/30 (53.3%) cases were selected during the dry cooler season *vs*. 26/60 (43.3%) controls (*p* = 0.37). Cases had a slightly lower body weight than controls (8.1 kg *vs*. 8.8 kg, *p* = 0.06), and malnutrition, measured by WAZ scores was significantly more prevalent in cases (70.0% *vs*. 41.7%, *p* = 0.01). Overall the two groups showed comparable distributions of values for the potentially confounding factors of age, sex, and recruitment season. Among cases, 23 presented bacteremic pneumococcal pneumonia, 2 had pneumococcal meningitis and 5 had bacteremia without a clear focus. Demographic and epidemiological characteristics of the study participants are shown in Table 1.

**Table 1:** Comparison of demographic and epidemiological characteristics in cases and controls.

Variable	Case Group (n = 30)	Control Group (n = 60)	*p* value
Age, months, Md (IQR)	12.8 (9.8–25.0)	11.7 (8.5–19.9)	0.37
Age group, infants	12 (40.0)	32 (53.3)	0.23
Gender, male	21 (70.0)	42 (70.0)	1.00
Recruitment during dry season	16 (53.3)	26 (43.3)	0.37
Recruitment during the pre-vaccine era	25 (83.3)	60 (100.0)	**0.003**
Weight, kg, Md (IQR)	8.1 (6.1–10.0)	8.8 (8.0–11.1)	0.06
Underweight, WAZ< -1SD	21 (70.0)	25 (41.7)	**0.01**

Data expressed in No. (%), unless otherwise specified.

Abbreviations: WAZ, weight-for-age z-score; Md, median; IQR, interquartile range; SD, standard deviation.

### Results of laboratory assays in cases and controls

NP pneumococcal carriage was highly prevalent in both groups: 28/30 (93.3%) in cases *vs*. 53/60 (88.3%) in controls (*p* = 0.71). The calibration curve for NP pneumococcal DNA quantification showed a linear behavior over six orders of magnitude, defining a linear range between 2.6•10^3^ and 2.6•10^8^ copies/mL. The correlation coefficient R^2^ was 0.997 and the slope of the standard curve was -3.572 resulting in an efficiency of 90.6% for real-time PCR. Both parameters fell within the acceptable limits previously established.

Among study samples, quantification of NP pneumococcal load by *lytA* real-time PCR showed a significant difference in mean load between cases and controls (7.0 log_10_ copies/mL *vs*. 5.8 log_10_ copies/mL, *p*<0.001). Capsular typing revealed that cases were more extensively colonized by invasive serotypes 1, 5 and 7F/A (46.4% *vs*. 3.8%, *p*<0.001) and more frequently co-colonized by different serotypes (84.0% *vs*. 39.0%, *p*<0.001). Considering all serotype carriage events separately in each group, the most frequent serotypes identified in cases were 7C/(7B/40) (27.3%), 5 (10.6%), 6A/B (9.1%), and 23F (9.1%), whereas for controls, serotype 6A/B predominated (19.7%), followed by 19F/C/B (14.8.%), 7C/(7B/40) (13.1%) and 14 (11.5%).

Comparatively, mean NP pneumococcal load of cases recruited after the introduction of the pneumococcal conjugate vaccine did not substantially differ from that of cases recruited in the pre-vaccine period (6.8 log_10_ copies/mL *vs*. 7.1 log_10_ copies/mL, *p* = 0.52). Half of the cases (50%) tested positive for HIV. HIV-positive cases did not present with a significantly higher mean pneumococcal load than those being HIV-negative (7.3 log_10_ copies/mL *vs*. 6.8 log_10_ copies/mL, *p* = 0.22). Of note, data of co-infection with HIV were not available from control samples. Malaria co-infection was detected in 2/30 (6.7%) of cases. Respiratory viral infection (mono- or multiple) was highly prevalent in both cases (14/30, 46.7%) and controls (17/60, 28.3%), but the difference was not statistically significant (*p* = 0.08). A total of 33 viruses were detected, 16 in cases and 17 in controls, with rhinovirus being the most predominant finding in both groups (n = 25, in 10 cases and 15 controls), followed by adenovirus (n = 5, four cases and one control), parainfluenza virus (n = 2, one case and one control), and influenza (n = 1, one case).

Median hemoglobin (Hb) levels were significantly lower in cases than in controls (74 g/L *vs*. 103 g/L, *p*<0.001), similarly to median hematocrit (HCT) values (23.5% *vs*. 31.0%, *p*<0.001). In contrast, no significant differences were found in median platelet (PLT) and white blood cell (WBC) counts. Table 2 describes results of laboratory microbiological and hematological tests in the two groups.

**Table 2:** Comparison of laboratory test results in cases and controls.

Variable	Case Group (n = 30)	Control Group (n = 60)	p value^a^^,^^b^
NP pneumococcal carriage	28/30 (93.3)	53/60 (88.3)	0.71^a^
NP lytA real-time PCR load, log10 cp/mL, mean (SD)	7.0 (0.9)	5.8 (1.0)	**<0.001**
NP pneumococcus serotype high invasiveness potential	13/28 (46.4)	2/53 (3.8)	**<0.001**
NP colonization with one serotype	4/28 (14.3)	25/53 (47.2)	**0.003**
NP co-colonization with 2 serotypes	8/28 (28.6)	12/53 (22.6)	0.56
NP co-colonization with 3 serotypes	6/28 (21.4)	4/53 (7.6)	0.09^a^
NP co-colonization with 4 serotypes	7/28 (25.0)	0/53 (0.0)	**<0.001**^a^
NP colonization with non-identified serotypes	3/28 (10.7)	12/53 (22.6)	0.19
Serotypes carried, Md (IQR)	3 (2–4)	1 (1–2)	**<0.001**^b^
PCV10 serotype coverage	25/28 (89.3)	31/53 (58.5)	**0.004**
PCV13 serotype coverage	25/28 (89.3)	38/53 (58.5)	0.07
HIV positive detection*	14/28 (50.0)	-	-
Malaria positive detection*	2/30 (6.7)	0/59 (0.0)	0.05^a^
Respiratory virus positive detection	14/30 (46.7)	17/60 (28.3)	0.08
Human rhinovirus	10/30 (33.3)	15/60 (25.0)	0.41
Adenovirus	4/30 (13.3)	1/60 (1.7)	**0.02**^a^
Influenza virus	1/30 (3.3)	0/60 (0.0)	0.16^a^
Parainfluenza virus	1/30 (3.3)	1/60 (1.7)	0.60^a^
Hb level, g/L, Md (IQR)* (cases: n = 29; controls: n = 57)	74 (66–99)	103 (94–110)	**<0.001**^b^
HCT, Md (IQR)* (cases: n = 29; controls: n = 57)	23.5 (21.5–29.4)	31.0 (28.6–33.5)	**<0.001**^b^
PLT, 10^3^ cell/mm^3^, Md (IQR)* (cases: n = 29; controls: n = 57)	421 (342–536)	494 (388–563)	0.83^a^
WBC, 10^3^ cell/mm^3^, Md (IQR)* (cases: n = 28; controls: n = 57)	14.4 (9.0–18.9)	12.8 (10.6–15.4)	0.17^a^

Data expressed as proportions (%), unless otherwise specified.

Abbreviations: NP, nasopharyngeal; Sp, *Streptococcus pneumoniae*; PCR, polymerase chain reaction; cp, copies; Md, median; IQR, interquartile range; SD, standard deviation; PCV, pneumococcal conjugate vaccine; HIV, human immunodeficiency virus; Hb, hemoglobine; HCT, hematocrit; PLT, platelet; WBC, white blood cell.

* Missing values

^a^ Fisher's exact test

^b^ Mann-Whitney test

### Factors associated with IPD

**Fig 1:**
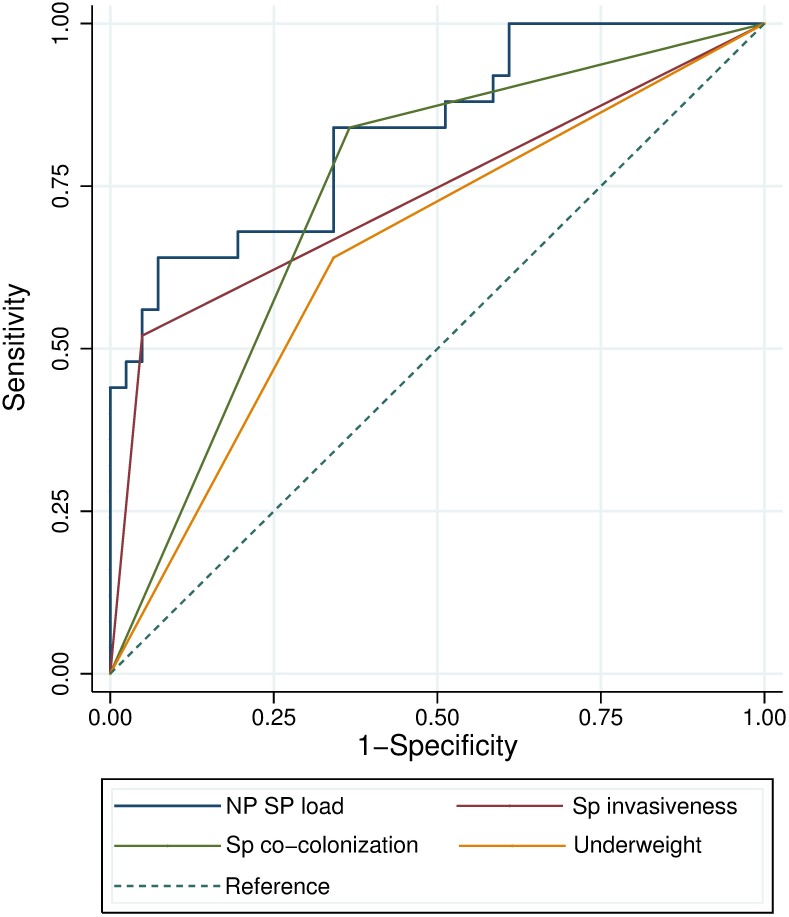
Accuracy of diagnostic predictors for IPD. Abbreviations: NP, nasopharyngeal; Sp, *Streptococcus pneumoniae*.

NP pneumococcal load quantified by *lytA* real-time PCR, NP pneumococci co-colonization, invasive serotypes, underweight, and Hb and HCT markers were significantly associated with IPD in the univariate analysis. Particularly NP pneumococcal load showed good discriminatory power between cases and controls (AUC-ROC: 82.1%, 95% CI 72.5%-91.8%). A colonization density of 6.5 log_10_ copies/mL was determined to be the optimal cut-off value to distinguish cases from controls, yielding sensitivity and specificity values of 75.0% and 73.6%, respectively. A fair diagnostic accuracy was observed for serotype invasiveness (AUC-ROC: 71.3%, 95% CI 61.6%-81.8%), pneumococcal co-colonization (AUC-ROC: 72.5%, 95% CI 62.0%-83.0%), and underweight (AUC-ROC: 64.2%, 95% CI 53.7%-74.6%), while Hb and HCT were poor predictors. Fig 1 depicts the ROC curves related to these risk factors comparatively.

Variables significantly associated with IPD identified in the univariate analysis were considered for the multivariate analysis. However, given the high co-linearity found between Hb and HCT (correlation coefficient *r* = 0.96, *p*<0.001) we decided to only include Hb in the modeling process as a more meaningful potential marker for IPD than HCT. A model including NP pneumococcal load, serotype invasiveness, and Hb proved to have the highest predictive power (AUC-ROC: 98.2%, 95% CI 80.8%-94.0%) and high levels of sensitivity (92.6%) and specificity (96.0%). The adjusted odds ratio for IPD per log unit increase of NP pneumococcal load was 8.06 (95% CI 1.57–41.41, *p* = 0.01) in this model. Results of univariate and multivariate analysis are presented in Table 3.

**Table 3:** Factors associated with IPD.

Variable	Univariate analysis			Multivariate analysis*		
	OR	95% CI	*p* value	Adjusted OR	95% CI	*p* value
NP lytA real-time PCR load, per 1 log_10_ cp/mL increase	3.83	1.98–7.41	**<0.001**	8.06	1.57–41.41	**0.01**
NP pneumococcus invasive serotype vs. non invasive	22.10	4.48–109.04	**<0.001**	134.82	5.88–3093.30	**0.002**
NP pneumococcal co-colonization vs. single colonization	8.20	2.37–28.3	**<0.001**			
Underweight vs. normal weight	3.27	1.28–8.32	**0.01**			
Respiratory virus positive detection vs. negative	2.21	0.89–5.50	0.09			
Hb level, per 1 g/L increase	0.92	0.88–0.95	**<0.001**	0.80	0.70–0.92	**0.002**
HCT, per 1% increase	0.75	0.66–0.86	**<0.001**			
WBC, per 10^3^ cell/mm^3^ increase	1.07	0.99–1.17	0.10			
PLT, per 10^3^ cell/mm^3^ increase	0.99	0.99–1.00	0.79			

*Selected model

Abbreviations: OR, odds ratio; CI, confidence interval; NP, nasopharyngeal; PCR, polymerase chain reaction; cp, copies; Hb, hemoglobine; HCT, hematocrite; PLT, platelet; WBC, white blood cell.

## Discussion

This study provides robust evidence for the suitability of pneumococcal bacterial load measured with a *lytA* real-time PCR in NP aspirates as a proxy of IPD diagnosis in children. NP pneumococcal load, once adjusted by a variety of potential host and pathogen risk factors, was significantly higher among cases than in healthy controls. We were able to identify a threshold value of 6.5 log_10_ copies/mL, which discriminated with moderately high sensitivity (75%) and specificity (73.6%) between cases and controls.

The idea of such a discriminating threshold is not new, and has previously been explored for diverse pneumococcal conditions, populations and sample types, but with unequal and conflicting results. A study by Anh et al. documented high rates of colonization density (≥6 log_10_ CFU/mL) measured by culture among NP swab samples from Vietnamese children with pneumonia [14]. A similarly high value of pneumococcal load (≈6 log_10_ copies/mL) quantified by PCR in NP samples was reported by Vu et al. for children with community-acquired pneumonia with radiological confirmation in the same country [15] while Fan et al. observed a slightly lower density (4.5–5 log_10_ copies/mL) for young Peruvian children with acute respiratory infection [16]. Interestingly, no threshold value for colonization density was established in these pediatric studies, probably because of the difficulty to establish such a value among children with disease and carriers, both of them having a high NP pneumococcal load.

In similar lines, Albrich et al. assessed the potential of quantitative *lytA* real-time PCR for the diagnosis of pneumococcal pneumonia in NP swabs from South African adults [17], determining a threshold value of 8,000 copies/mL with a sensitivity of 82.2% and a specificity of 92.0%. In a subsequent study on the same population, they proposed a similar cut-off value of 10,000 copies/mL for good-quality sputum *lytA* real-time PCR, albeit with slightly lower sensitivity and specificity values (78.1% and 80.0%, respectively), to discriminate HIV-infected adults with pneumococcal pneumonia from controls [18]. Stralin et al. documented cut-off values of 100 copies/mL for NP aspirates and 4.5 log_10_ copies/mL for sputum measured by quantitative *lytA* real-time PCR in an elder Swedish pneumonia cohort [19]. Use of real-time PCR detection on sputum samples was also postulated by Yang et al. for rapid diagnosis of adult CAP pneumonia in an Emergency Department [20]. This group determined an AUC of 0.87 under the ROC curve, reporting sensitivity and specificity values of 90% and 80% respectively for a cut-off of 37,000 copies/mL.

We speculate that the ≥ 2-log difference in the cut-off value between these studies on adult populations and ours could primarily be due to the impaired immune response during first years of life related with the high rates of carriage in children [32], in addition to different sampling and quantification methods and epidemiological characteristics. Given the high densities of pneumococcal carriage (up to 8.5 log_10_ copies/mL) found in cases we estimate that the relatively high threshold value calculated in our study (6.5 log_10_ copies/mL) may have diagnostic utility to diagnose pediatric IPD.

A trend for a synergistic relationship in the univariate analysis between respiratory virus co-infection and IPD was also identified but did not reach statistical significance. This outcome is in disagreement with strong synergistic associations previously described between pediatric pneumococcal pneumonia and viral co-infection [15] and between pediatric invasive pneumococcal pneumonia and rhinovirus co-infection [33]. We hypothesize that the low prevalence of influenza in our study population and the relatively stable incidence of rhinovirus infection throughout the entire study period could explain that respiratory viral co-infection was not found to be a risk factor.

In recent years, technological advances have allowed integration of molecular diagnostics into small footprint devices, which perform fast, simple, highly sensitive molecular assays at the point of need. In the light of these diagnostic innovations, the potential implications and applicability of our findings are promising, particularly for pediatric populations, who would also benefit from the high diagnostic yield of NP washed in addition to the convenience of the sampling method. As a novel biomarker, NP pneumococcal DNA load may allow designing point-of-care tests capable of quantifying bacterial load in a simple manner. In addition, the results of the multivariate analysis assessing independent risk factors for IPD suggest that the adoption of a holistic approach by combining quantification of NP pneumococcal load with serotype identification, and quantification of blood markers could substantially increase overall diagnostic yield. Such comprehensive approach would especially be suitable in reference healthcare settings with laboratory infrastructures equipped to routinely perform all these techniques.

This study has various important limitations. First, our total sample size was small, although the analysis seems to have been sufficiently powered to show real differences in NP pneumococcal load between cases and controls, and the differences observed between groups are biologically plausible to prove the concept. Second, in spite of the fact that a case-control approach appears suitable for the testing of the project’s hypothesis, restricting the selection of controls to a short period of time once all cases had been recruited could have introduced important biases in our results, particularly related to variability in patient’s characteristics, or with year-to-year variations of the different infections (bacterial and viral) assessed, or the incidence of infecting pneumococcal serotypes. A comparability analysis was performed to control the potential confounding effect of seasonality on NP colonization density. However the small sample size hinders our capacity to evaluate year-to-year variation of circulating serotypes. Third, HIV status in controls was unknown but we have no reason to suspect that it could be considerably different from that reported for healthy children in the same community, for whom a low HIV prevalence in a range of 3–5% has been documented [12]. Moreover, when we performed a sub-analysis of cases there were not significant differences in mean NP pneumococcal load between HIV co-infected and not co-infected subjects, which suggests that this factor does not substantially increase NP density of pneumococcus in young patients. Finally, these results are drawn from a population with very high background prevalence rates of nasopharyngeal pneumococcal carriage, as previously described, and it remains to be seen whether the threshold obtained in our study would remain valid in areas with lower carriage rates [34].

## Conclusion

In conclusion, we propose a new diagnostic method which couples use of easily obtainable non-invasive NP aspirates with rapid and accurate quantification of NP pneumococcal load by *lytA* real-time PCR as a surrogate marker for IPD in children. Further validation is required to confirm the clinical utility of this method.

## Supporting information

S1 Dataset Supporting data for the manuscript results. (XLS)
